# Determination of lipid requirements in black soldier fly through semi-purified diets

**DOI:** 10.1038/s41598-022-14290-y

**Published:** 2022-06-28

**Authors:** S. Bellezza Oddon, I. Biasato, A. Resconi, L. Gasco

**Affiliations:** Department of Agricultural, Forest and Food Sciences, University of Turin, Turin, Italy

**Keywords:** Physiology, Zoology

## Abstract

The insect market is still far from an effective upscale and, to achieve this goal, it is necessary to know the BSF dietary requirements for the production maximization. Worldwide, given the waste variability, is not always easy to identify the optimal waste-based mixture that can allow to reach the best production, in terms of quantity and quality. Due this reason, nutritional need ranges are the basic knowledge, affordable for everyone, to increase the profitability of the insect farming. The study aims to evaluate the effects of 6 semi-purified, isonitrogenous and isoenergetic diets (SPII) with increasing lipid levels (1%, L1; 1.5%, L1.5; 2.5%, L2.5; 3.5% L3.5; 4.5%, L4.5) on BSF life history traits (6 replicates/treatment and 100 larvae/replicate). The Gainesville diet was used as environmental control. Considering the whole larval stage, 4.5% lipid level guarantees better performance when compared to content lower than 2.5%. The L4.5 10-day-old larvae yielded greater when compared to the other dietary treatments. At 14 and 18 days of age, the larvae of the groups above 2.5% performed better than L1, while the L1.5 showed intermediate results. Lipid levels below 1.5% on DM, when compared to 4.5%, resulted in a smaller prepupa and pupa size. The results obtained on the adult stage do not allow the identification of a lipid levels ideal range, as in the larval stage. In conclusion, in the whole larval stage and in prepupae/pupae phases, lipid percentage lower than (or equal to) 1% have a negative effect on growth. Other research will be needed in order to evaluate lipid levels above 4.5% on DM.

## Introduction

Recently, insects are receiving more and more attention as farmed animals, both for feed and food production. Their capacity to be reared using less space and water, their low greenhouse gases emissions, and their high protein content make them a valuable and sustainable choice as alternative sources of protein^1,2^. Out of the 7 species allowed to be farmed for poultry, swine and aquaculture feed production, the black soldier fly (BSF, *Hermetia illucens*) is the one who is receiving the most attention from both the scientific community and the industry sector^3^. Its cosmopolitan distribution, the ability to feed on a large variety of organic substrates, the relative ease of rearing compared to other insect species, and its high feed conversion rate make the BSF one the most important candidates as novel protein source for the feed sector^4–6^. Black soldier fly larvae (BSFL) can, indeed, be reared on waste that would be otherwise sent to landfills or, in the best-case scenario, composting facilities^7,8^. Composting, however, would not valorise the remaining nutrient content of the organic material that is used for it. The BSFL breeding, on the other hand, can turn waste into valuable protein for the feed industry^9,10^. The insect protein industry is currently facing two main challenges that still prevent it to become competitive on the market: the need to upscale and the regulatory barriers^11^. Despite the recent authorization of BSFL-based protein feed for poultry and pigs (Reg. (EU) 2021/1372) having allowed to significantly extend the legislative framework, the insect market is still far from an effective upscale^12–14^.

The necessity of insect industrial production maximization stimulates the scientific community to pursue its efforts on BSFL rearing requirements determination, and the knowledge of bred-animals nutritional needs is the farming optimization starting point. This has been known for decades for the major animal species reared for human consumption, but, in spite of the high number of studies done on BSF, there is still a very limited knowledge about the dietary requirements for the correct development of the larvae and for their metamorphosis into imagos (adult stage^15–17^). The scientific works currently available in literature focus on protein and carbohydrate needs, as well as the relationship between the two^18,19^. Proteins represent an important nutritional source, since they have a plastic and growth-related function, while carbohydrates are an energetic font. Since the holometabolous insect (they present a larval stage and an adult stage with clear differences: feeding habits, morphology) alternates in different development phase feeding and non-feeding periods, the fat body cells are necessary for their survival during starvation^20^. During the feeding-phase, the carbohydrates surplus is converted in lipid reserve thanks to the insulin activity^1,21^.

The lipid component has another important function during the Diptera life cycle. In particular, the moulting process begins from the ecdysteroids, which are hormones that belong to the steroid class and whose synthesis occurs from the cholesterol ingested in the feeding phase^22^. In a recent paper by Bellezza Oddon et al.^23^, a reduced fly emergence rate was observed in the experimental diets with halved lipid content (ether extract [EE] as is: < 1.27) when compared to the GA diet (EE as is: 2.22). In arthropod, the moult occurs thanks to steroid hormones called ecdysteroids. Insects cannot synthesize cholesterol and, consequently, the feeding-phase is important for its assimilation^24^. Another study reported the important role that lipids play in the development of BSFL, with longer time needed to reach the prepupal stage in diets lower in lipids and vice-versa^25^.

Since lipids seems to have an effect on insect physiology, can they also have an influence on growth and to what extent?

Based on the above-reported background, this study aims at assessing the optimal lipid content that can guarantee the greatest development of BSF throughout their entire cycle, from larval stage to pupation and fly emergence by using semi-purified, isonitrogenous and isoenergetic diets.

## Results

### Larval stage

**Figure 1:**
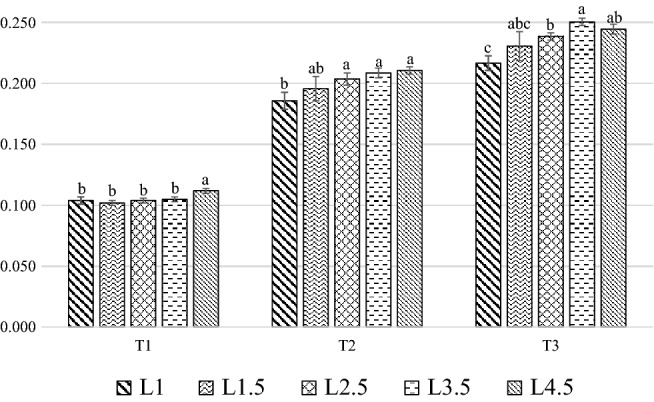
Effects of the interaction between dietary treatment and time on larva growth. *L1* EE 1% diet, *L1.5* EE 1.5% diet, *L2.5* EE 2.5% diet, *L3.5* EE 3.5% diet, *L4.5* EE 4.5% diet, *GA* Gainesville diet, *T1* 10 day old, *T2* 14 day old, *T3* 18 day old. Means with different superscript letters (a, b, c) within the same row differ significantly (*p* < 0.05). The standard error is represented by the error bars.

At 6 days of age, the larvae weight was analogous in all the treatments (0.088 g ± 0.001; *P* > 0.05). Table 1 illustrates the effects of the diet and the time on larvae growth. Considering the whole larval stage, the treatment with the highest lipid content (L4.5) showed heavier weight when compared to the L1, L1.5 and L 2.5 diets (0.180 g, 0.161 g, 0.167 g and 0.172 g, respectively; *P* < 0.01), while the L3.5 group (0.176 g) displayed an intermediate result between the L2.5 and the L4.5 (*P* > 0.05). Time had, naturally, an effect on larvae weight (T1, 10 days old; T2, 14 days old; T3, 18 days old; *P* < 0.01). The interaction between diet and time is shown in Fig. 1. At T1, the L4.5 treatment performed better (0.112 g) when compared to the other groups (L1, 0.104 g; L1.5, 0.102 g; L2.5, 0.104 g; L3.5, 0.105 g; *P* < 0.01). In the second sampling time (T2), the L2.5 and L3.5 treatments were not different from the L4.5 (0.204 g, 0.209 g and 0.211 g, respectively; *P* > 0.05). On the contrary, the L1 larvae showed the worst result (0.186 g; *P* < 0.01), while L1.5 did not differ among the groups (0.196 g; *P* > 0.05). At T3, the L3.5 group showed higher larvae weight than the L1 and the L2.5 (0.251 g, 0.217 g and 0.239 g, respectively; *P* < 0.01), but analogous results in comparison with the L1.5 and the L4.5 treatments (0.231 g and 0.245 g; *P* > 0.05). Regarding the survival rate, no differences were observed among the groups (L1, 85%; L1.5, 93%; L2.5, 90%; L3.5, 95%; L4.5, 95%; *P* > 0.05).

**Table 1:** Effects of the dietary treatment, time and interaction between dietary treatment and time on individual larval weight.

Parameter	Diet (D)					Time (T)			SEM		*p*-value		
	L1	L1.5	L2.5	L3.5	L4.5	T1	T2	T3	D	T	D	T	DxT
Weight (g)	0.161^d^	0.167^ cd^	0.172^bc^	0.176^ab^	0.180^a^	0.105^c^	0.201^b^	0.236^a^	0.003	0.002	0.001	0.001	0.001

L1 EE 1% diet, L1.5 EE 1.5% diet, L2.5 EE 2.5% diet, L3.5 EE 3.5% diet, L4.5 EE 4.5% diet, T1 10-day-old, T2 14-day-old, T3 18-day-old, SEM standard error of the mean.

Means with different superscript letters (a, b, c) within the same row differ significantly (*p* < 0.05).

### Chemical analysis

The descriptive chemical composition of the larvae is shown in Table 2. The DM ranged from 29.76 to 32.43%. The percentage on the larvae DM of the EE tend to decrease with the increasing of the diet EE level (L1:47.45%, L1.5: 39.39%, L2.5: 38.52%, L3.5: 37.91% and L4.5: 33.21%). The GE values, CP and ash content on DM ranged from 28.97 MJ/kg to 30.14 MJ/kg, from 23.11% to 30.35% and from 1.69 to 1.87%, respectively.

**Table 2:** Larvae descriptive chemical composition (g/100 g on DM) and gross energy (MJ/kg on DM) expressed on DM. L1 EE 1% diet, L1.5 EE 1.5% diet, L2.5 EE 2.5% diet, L3.5 EE 3.5% diet, L4.5 EE 4.5% diet, DM dry matter, CP crude protein, EE ether extract, GE gross energy.

Items^a^	Larvae (% on DM)				
	L1	L1.5	L2.5	L3.5	L4.5
DM	32.43	30.80	29.76	31.40	30.11
CP	23.11	28.44	30.44	28.28	30.35
EE	47.45	39.39	38.52	37.91	33.21
Ash	5.22	5.61	5.77	5.95	6.16
GE	30.14	29.16	28.95	28.97	29.58

^a^Values are reported as mean of duplicate analyses.

### Prepupae

**Figure 2:**
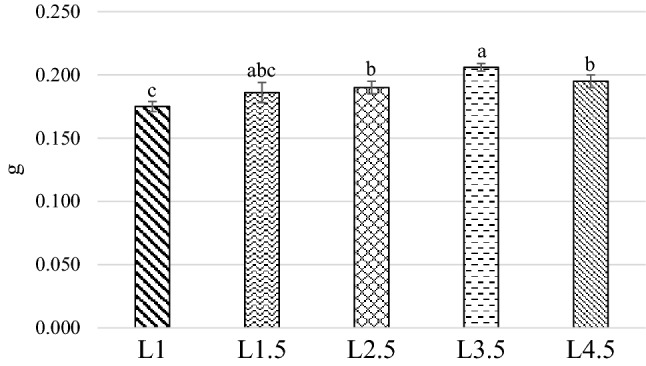
Diet effect on the prepupae weight. *L1* EE 1% diet, *L1.5* EE 1.5% diet, *L2.5* EE 2.5% diet, *L3.5* EE 3.5% diet, *L4.5* EE 4.5% diet, *GA* Gainesville diet. Means with different superscript letters (a, b, c) within the same row differ significantly (*p* < 0.05). The standard error is represented by the error bars.

**Figure 3:**
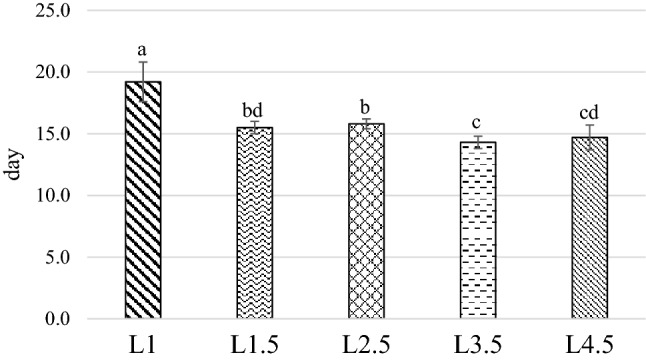
Time necessary to reached the prepupae phase. *L1* EE 1% diet, *L1.5* EE 1.5% diet, *L2.5* EE 2.5% diet, *L3.5* EE 3.5% diet, *L4.5* EE 4.5% diet, *GA* Gainesville diet. Means with different superscript letters (a, b, c) within the same row differ significantly (*p* < 0.05). The standard error is represented by the error bars.

Figure 2 and 3 summarizes the diet effect on prepupae weight and the L-Pp development time. The L1 group showed a lower weight when compared to the L2.5, L3.5 and L4.5 diets (0.175 g vs 0.190, 0.206 and 0.196, respectively; *P* < 0.001), with L3.5 being the one which yielded the heaviest prepupae (*P* < 0.001). The L1.5 treatment was not significantly different from the other experimental diets (0.186 g; *P* > 0.05).

The L1 treatment had the longest development time (19.2 days), while the diet which caused the fastest development was the L3.5 (14.3 days), which was not significantly different from L4.5 (14.7 days; *P* > 0.05). The L2.5 prepupae showed an intermediate result between the L1 and L3.5 (15.8 days; *P* < 0.001). Finally, the L1.5 group (15.5 days) performed similarly when compared to L2.5 and L4.5 (*P* > 0.05).

### Pupal stage

**Figure 4:**
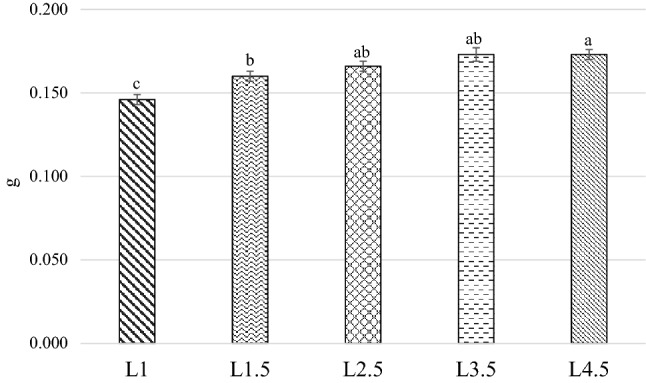
Effect of the diet on the pupae weight. *L1* EE 1% diet, *L1.5* EE 1.5% diet, *L2.5* EE 2.5% diet, *L3.5* EE 3.5% diet, *L4.5* EE 4.5% diet, *GA* Gainesville diet, *T1* 10 day old, *T2* 14 day old, *T3* 18 day old. Means with different superscript letters (a, b, c) within the same row differ significantly (*p* < 0.05). The standard error is represented by the error bars.

**Figure 5:**
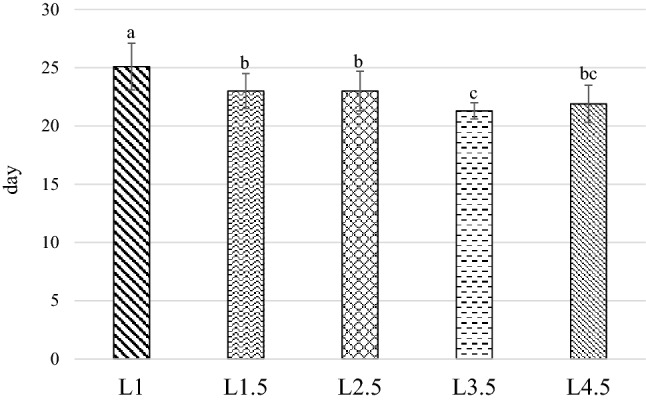
Larva-pupa duration time (day). *L1* EE 1% diet, *L1.5* EE 1.5% diet, *L2.5* EE 2.5% diet, *L3.5* EE 3.5% diet, *L4.5* EE 4.5% diet, *GA* Gainesville diet, *T1* 10 day old, *T2* 14 day old, *T3* 18 day old. Means with different superscript letters (a, b, c) within the same row differ significantly (*p* < 0.05). The standard error is represented by the error bars.

Data about pupae weight and L–P duration time are illustrated in Figs. 4 and 5. The pupae of the L4.5 diet were heavier than the L1 and L1.5 groups (0.173, 0.146 g and 0.160 g, respectively; *P* < 0.001). The L2.5 (0.166 g) and L3.5 (0.173 g) pupae displayed similar weights to L4.5 and L1.5 treatments (*P* > 0.05). The L–P time duration showed an opposite trend when compared to the weight, as the L3.5 treatment developed faster than the L1, L1.5 and L2.5 groups (21.3 days, 25.1 days, 23 days and 23 days, respectively; *P* < 0.01), but was not significantly different from L4.5 (21.9 days; *P* > 0.05). The L4.5 also displayed analogous L–P duration times to those of the L1.5 and L2.5 diets (*P* > 0.05), while the L1 group had the slowest development when compared to the other groups (*P* < 0.01).

### Adult stage

Table 3 illustrates the effect of the diet and the sex on the adult parameters. The longest FLS was recorded in the L2.5 treatment and the shortest in the L1.5 group (8.66 days and 7.90 days, respectively; *P* < 0.05), while the L1, L3.5 and L4.5 did not differ from the other experimental diets (*P* > 0.05). The FLS was also influenced by the sex, as the males had a longer lifespan when compared to the females (*P* < 0.001). The WR was also affected by the treatment and sex. During the life of the flies, the L1, L1.5 and L4.5 showed a greater WR than L3.5 (*P* < 0.05), while L2.5 had analogous WR to all the treatments (*P* > 0.05). As regards the sex effects on WR, the females lost more weight during the lifetime than the males (*P* < 0.01). The FLW and P–F duration time were affected by both the treatment and the sex, while the PW only by sex. The L3.5 FLW was numerically heaviest and significantly different from L1 and L1.5 (*P* < 0.01). No differences were observed among the FLW of L1 and L1.5, while the L2.5 and L4.5 only varied from L1 (*P* < 0.01). The P–F duration time was shorter in the L1, L1.5 and L2.5 diets when compared to the L4.5 group (*P* < 0.05). On the contrary, the L3.5 diet did not differ from the other dietary treatments (*P* > 0.05). Considering the sex influence, females took longer time to become flies and displayed a greater FLW and PW when compared to the males (*P* < 0.05). Finally, the ER and SR were not influenced by the dietary treatments (Table 4; *P* > 0.05).

**Table 3:** Effects of the diet and sex on adult parameters.

Parameters	Diet (D)					Sex (S)		SEM		*p*-value	
	L1	L1.5	L2.5	L3.5	L4.5	F	M	D	S	D	S
FLS (day)	8.09^ab^	7.90^b^	8.66^a^	8.31^ab^	8.28^ab^	7.48	9.08	0.200	0.115	0.022	0.000
WR (%)	51.46^a^	52.29^a^	51.43^ab^	50.37^b^	51.67^a^	52.09	50.80	0.453	0.287	0.039	0.001
FLW (g)	0.082^c^	0.087^bc^	0.095^ab^	0.101^a^	0.093^ab^	0.102	0.081	0.003	0.002	0.001	0.000
P–F (day)	6.98^b^	7.14^b^	7.11^b^	7.18^ab^	7.45^a^	7.26	7.07	0.063	0.125	0.015	0.016
PW (g)	0.021	0.025	0.024	0.027	0.025	0.026	0.023	0.002	0.001	0.263	0.000

D diet, S sex, F female, M male, L1 EE 1% diet, L1.5 EE 1.5% diet, L2.5 EE 2.5% diet, L3.5 EE 3.5% diet, L4.5 EE 4.5% diet, SEM standard error of the mean, FLS fly lifespan, WR weight reduction, FLW fly live weight, P–F pupae fly duration time, PW puparium weight.

Means with different superscript letters (a, b, c) within the same row differ significantly (*p* < 0.05).

**Table 4:** Effect of the diet on the emergence rate and sex ratio.

Parameters	Diet					SEM	*p*-value
	L1	L1.5	L2.5	L3.5	L4.5		
ER	87.70	88.83	94.16	95.99	95.76	3.154	0.083
SR	0.77	0.93	0.95	0.86	0.85	0.123	0.734

L1 EE 1% diet, L1.5 EE 1.5% diet, L2.5 EE 2.5% diet, L3.5 EE 3.5% diet, L4.5 EE 4.5% diet, ER emergence rate, SR sex ratio, SEM standard error of the mean.

## Discussion

### Larval stage

In recent years, research has started to focus the attention on the BSF nutritional requirements determination. In particular, studies related to protein, energy and carbohydrate needs have recently been carried out^23,26,27^. The knowledge on BSF nutrient requirements is still limited, even if the great number of scientific papers published on its life cycle and its use as feed ingredient^28–30^. In insects with a non-feeding adult stage, the ratio between protein and carbohydrate plays a significant role for the survival, growth and reproduction^19^. On the other side, lipids are an important source of energy during metamorphosis^30^, and, to the author’s knowledge, data about the effect of the substrate lipid content on BSF life history traits are absent in literature.

Since the weight gain and development time of the larvae reared on the GA diet presented a growth pattern in line with those regularly recorded in the experimental centre colony (data not shown), it can be excluded that some environmental factors altered the results of the trial.

Overall, the difference between the treatments with the lowest and highest lipid level underlines that not only the protein and the carbohydrate content have an effect on the growth performance of the larvae. Despite Barragan-Fonseca et al.^19^ having observed that the larvae yield was greater in the diets with a high amount of carbohydrate (NSC percentages on DM: 35, 45 and 55), in the present study, the better growth was recorded in the treatment with the lowest carbohydrate content (76.33% of NSC on DM) when compared to the other treatments (NSC range: from 77.87 to 80.17% on DM). Furthermore, since the protein level was equal in all the experimental diets, it may be possible to attribute the difference in growth between L1 and L4.5 to the lipid effect.

Considering larval stage at different times, at T1 L4.5 yielded the biggest larvae when compared to the other dietary treatments. At 14 and 18 days of age, the larvae of the groups above 2.5% performed better than L1, while the L1.5 showed intermediate results. Since the L1.5 treatment showed great variability, no difference was observed among the L1.5 treatment and the others. The human error was excluded as an influencing factor as the standard error value maintained high at T2 and T3, as well as for the prepupae weight. From a nutritional point of view (lipid and protein content), the L3.5 dietary treatment is partially comparable to the ED_1_ diet formulated by Georgescu et al.^26^. Already at 10 days of age, the L3.5 larvae were smaller than ED_1_ (0.105 mg and about 0.162 mg, respectively). The difference between the two studies may be due to the different carbohydrate percentage or to the quality of the supplied macronutrients. In particular, in semi-purified diets macronutrients are present almost in purity when compared to the ingredients of GA and soybean meal used by Georgescu et al.^26^. Finally, considering that the marked difference recorded at T1 among the treatment with the highest lipid content and the others was lost at T2 and T3, it is possible to hypothesize that the lipid requirement is highest in early larval stages. A similar trend was observed in the results from the Bellezza Oddon et al.^23^ study, where larval weight at 10 days was highest in the treatment with the highest percentage of CP (19% of DM), while at 18 days the treatment that yielded the best results in terms of weight was 14% CP on DM. This can point out that in early stages of development larvae have greater needs in terms of nutrients and during the latest stages those needs diminish. In the present trial, no differences can be observed between 2.5% and 4.5% at 14 and 18 days of age. In the light of this result, it could be interesting, in an industrial rearing facility, to tailor specific diets for the first stages of development that are rich in nutrients, and use substrates that are less nutritious for the late stages. Similar results were found by Meneguz et al.^32^ between larvae reared on brewery by-products (BRE) and on winery by-products (WIN). The BRE diet had higher EE and CP (respectively 86.7 and 200.5 g/kg of DM) when compared to the WIN diet (79.0 and 117.4 g/kg of DM). At 10 days of age, the BRE larvae yielded the best results than WIN (92 mg and 17 mg, respectively), while at the end of their development there was no significant difference between the two treatments. Also in this case, a substrate richer in nutrients impacted significantly on BSFL early stages growth, while differences in the later instars are less evident.

Larval survival among the treatments was not different, and the percentages of survivorship from larvae to prepupae are similar to the values found by Tomberlin et al.^33^.

The lipid composition of the L1.5, L2.5 and L3.5 larvae is in agreement with the range from 37 to 41% of EE on DM observed by Georgescu et al.^26^ in the diet with about 3% of EE and 13.5% of CP. On the contrary, L1 had the highest fat content (47.5% on DM) and L4.5 the lowest one (33.2% on DM). The larvae fat fraction decreases with increasing the lipid content of the diets. In particular, when compared to the L4.5 larvae, the L1 were composed of 30% more lipids. The presence of a greater amount of fat in the larvae with a low-fat diet can be explained by the transformation of the excess carbohydrates into a lipid reserve^34^. To maintain diets isoenergetic, the amount of carbohydrates was reduced as the lipid level increased. Cohn et al.^27^ evaluated the effect of different carbohydrates groups on the proximate composition of the larvae and observed that, in diets containing wheat starch, the lipid percentage of the larvae was higher than the corn starch (over 45% on DM). Regarding the protein content of the larvae, all the dietary treatment showed lower values (≤ 30% on DM) when compared to those observed by Gold et al.^35^ [> 33% on DM]) in waste-based diets with 14% of CP and over 5% of EE on DM. Although the protein level of the experimental diets was the same, a difference was found in the protein composition of the larvae. In particular, the difference in percentage between the treatments with the highest (L4.5) and lowest (L1) values was 7.24%. Considering the same protein level in the diets and the difference in the composition of the larvae, it is possible to hypothesize that the metabolization of the protein content is a complex physiological mechanism and may derive from macroelement interactions. As regard the larvae ash content, although the experimental diet had the same percentage, in the larvae the quantity of minerals increases with increasing the lipid level. This nutritional fraction of the diet is currently little-studied, but the metabolization of micronutrients can have an influence on the quality of the product derived from the BSF larvae breeding.

### Prepupal stage

If the variability of the L1.5 is considered as in larval stage, the L1 treatment yielded the smallest prepupae among all the treatments. In addition, the group with the lowest lipid content took the longest time to develop into prepupae. The L3.5 treatment had the best results in terms of weight, but, also in this case, the high SEM of the L1.5 treatment did not allow obtaining a statistical evidence. In terms of development time, the L3.5 performed best when compared to the treatments with lower lipid levels. In Arthropoda, if the nutritional requirements are not met, in quality or/and quantity, the development time can vary^24^. In the present study, the treatment with a lipid content greater than or equal to 1.5% on DM performed the shortest development time. Therefore, a reduced quantity of lipids can determine an increase in larval stage duration. The prepupae weight and the L–P duration time recorded in the present study differ from the results obtained by Tomberlin et al.^33^. The L1 group, the least performing, had an average weight higher than the best treatment of Tomberlin et al.^33^. Time needed to reach prepupal stage was also shorter in this study when compared to that was found by Tomberlin et al.^33^. Since the nutrient composition in the two studies was similar, these differences can be attributed to different breeding methods rather than to the diets. In particular, in the study conducted by Tomberlin et al.^33^, daily feeding was applied in opposition to the one-time feeding of the present trial.

### Pupal stage

Similarly to the prepupa phase, the L1 treatment showed the lowest weight and longest development time. Lipid levels below 1.5% on DM, when compared to 4.5%, resulted in a smaller pupa size (as a logical consequence of the lower larval and prepupal weights). Since the metamorphosis of the adult occurs in the puparium, a small pupa generates proportionately equal adults. In the three isonitrogenous and isolipidic diets with increasing metabolizable energy (from 2174.5 to 3044.5 kcal/kg) formulated by Georgescu et al.^26^, no difference in pupal weight was found. From a reproductive point of view, therefore, defining the optimal lipid level in the larval stage could allow for greater performance in the adult stage. Considering the development time, as stated in the previous sub-paragraph, the larval stage is the most important phase for the nutrient accumulation. For this reason, the longest development time of L1 at the prepupae stage affected, logically, the pupa's time to reach.

### Adult stage

Since the flies lived in a box with no intraspecific interaction and with a reduced energy consumption due to the low possibility of movement, the experimental conditions of the adult phase are not comparable to the breeding ones. However, considering that all the experimental treatments were subjected to the same environmental effects, the results obtained are attributable to the diets. The experimental diets influenced the FLS, and data recorded during the trial are partially in agreement with those of Bellezza Oddon et al.^23^, in which the diet with EE values close to 1% (CP14, CP16 and CP19) lived up to one day longer than L1 (8.43, 8.45, 9.36 days versus 8.05 day). Chia et al.^36^ and Qomi et al.^37^ observed the flies’ longevity at different temperature, and at 30 °C the average lifespan lies within the 5–10 days range – period comparable to the FLS of the semi-purified diets. Since the major numerical WR was manifested by the group with the least longevity (L1.5) and there is no clear relationship trend between the two variables, it is possible to hypothesize that weight loss is not influenced by FLS. The FLW was greater in the dietary treatment with a lipid level above 3.5% on DM when compared to L1 and L1.5 groups. Although there is no statistical difference, on a numerical level it is observable that diets below 1.5% of EE had a reduced weight when compared to the others. The live weight parameter varies widely even if related with similar diets in term of nutritional composition. For example, Georgescu et al.^26^ observed that the weight of flies derived from larvae fed on substrates with about 13.5% of CP and 3% of EE on DM lies in a range between 0.028 and 0.039 g, thus being much lower than the ones recorded in the present study (range: 0.082–0.101 g). Since in literature there are few studies concerning nutrients intake effects on the flies, it is difficult to hypothesize an exclusive diet influence—but more probably an interaction between diet and genetic.

As reported by Bellezza Oddon et al.^23^, the protein content in the diet does not influence the P–F duration time. On the contrary, the lipid percentage seems to have an effect on the intra-puparium development time. In particular, P–F time tended to get longer as the lipid level increased. The intra-puparial development can be influenced by several factor like temperature, pupation site and feed ingested during larval stage^34^. As regard the feed ingestion, the provision of inadequate feed can speed up this phase of BSF life cycle^34^. For this reason, it is possible to state that the L4.5% was a more complete diet since took longer P–F time when compared to diets with a lipid percentage below 2.5%. Few nutritional reserves combined with rapid development is a maximization of resources and increases, in case of unfavourable conditions, the probability of survival.

No differences were observed for the PW that was, logically, numerically lower in L1, the diet with the smaller flies, and higher in L3.5, in which the flies were bigger. Finally, the lipid percentage of the diet does not influence the ER, differently from what was assumed by Bellezza Oddon et al.^23^. Moreover, the sex ratio is not affected by the treatment, and in all the experimental diets the ratio was in favour of males as observed by Barragán Fonseca et al.^38^ in a vegetable-based diet with 10% of CP and 5.7% of EE on DM.

All the adult parameters analysed in the current study were affected by the sex. Data about the effect of the sex on the pupae development are still very limited. In the present study, the P–F duration time was longest in females. In contrast, no significant differences between sex in the pupae development were noted by Tomberlin and Sheppard^39^. Since the female spends more time in larval stage, it is possible to speculate that the same condition may occur in pupal phase, in which the formation of female reproductive system requires the longest period^40^. As noted by Bellezza Oddon et al.^23^, the fly sex had an effect on the life span, with the males living longer than females. Tomberlin et al.^41^ observed that males lived 3.4 d or 3.5 d longer than females at 27 °C and 30 °C, without evident temperature effects. In the present study, male’s lifespan was 1.6 d longer compared to females, and the difference between the two outcomes could be attributed to the nutrient accumulation during the larval stage and/or to genetics. The reduced lifespan of the females may be explained as a procreator mechanism. Since females did not display any multiple mating attempts during Jones et al.^42^ observations, it is possible to hypothesize that males mate more times than females and, consequently, a longer life span can allow it to have more fecundation success. In some insect species, the female mating occurs only once in the course of life, as, for example, many mosquitoes appear to be monandrous^43^. In other cases, as for *Epiphyas postvittana* (Walker; *Lepidoptera*: *Tortricidae*), the multiple mating of females is generally attributed to a male dysfunction^44^. As known in literature, the fly and the puparium weight are sex-related: females have a greater weight than males and, logically, also the puparium size follows the same trend^41^. The sex-linkage between lifespan and weight can be explained as an adaptative system derived from sex-specific optimization of reproduction and survival trade-off^45^. In particular, the highest female weight may derive from the necessity to accumulate a consistent amount of nutrients in the larval stage to produce eggs^41^. On the contrary, males live longer but have the lowest weight in order to have the most time possible to fecundate. Despite the shorter lifespan of the female, the percentage weight loss of the fly was greater in females than in males. It is possible to assume that the higher energy accumulated in larval stage by the female for egg production is consumed also in male absence. Indeed, it was hypothesized that, if the flies do not have the opportunity to mate early after the emergence, the oocytes are reabsorbed in order to sustain other vital functions^39^. Therefore, the female reduced clutch size (oocytes) may be the cause of the increased weight loss compared to the male.

## Conclusion

The determination of the macronutrients needs in the diets of the BSF represents one of the basis for the production maximization. As regards the lipids, in the whole larval stage and in prepupae/pupae phases, percentages lower than or equal to 1% of EE on the DM have a negative effect on growth (smallest size and longest development time). In the larvae earlier stages (up to 10 days), however, a lipid level equal to 4.5% allows obtaining better growth performance.

Based on this outcome, it is possible to hypothesize a greater lipid requirement during the first stages of growth, which tends to be less evident in the later larvae instars. The results obtained at the adult stage do not allow identifying an optimal diet at this level.

To the authors knowledge, the present study is the first one on the BSF lipid requirement assessment. Considering the reduced lipid content in the waste, low fat content levels has been tested. In addition, in order to maintain isoenergetic diets, only the range between 1.5 and 4.5% was evaluated. Since the results obtained are the inception of the optimal lipid level determination, further researches will be needed to evaluate the effect on BSF life history traits of percentage above 4.5% of EE on DM.

## Materials and methods

### Experimental diets

Five experimental diets with increasing percentage of lipids on DM, namely: 1% (L1), 1.5% (L1.5), 2,5% (L2.5), 3,5% (L3.5) and 4.5% (L4.5) were tested. The Gainesville diet (GA)^22^, which is normally used in the experimental facility (Tetto Frati—Department of Agricultural, Forest and Food Sciences; University of Turin) for the colony maintenance, was used as environmental control diet to assess the non-anomalies occurrence during the experiment and the quality of the larval batch. Therefore, data collected from this diet were not used for the statistical analysis. To prepare the GA, corn seeds and alfalfa pellets were ground in powder with particles smaller than 2 mm using a grinding mill (CL/5 Fimar, Italy) and successively mixed with wheat bran according to Hogsette^46^.

The semi-purified ingredients (wheat starch, casein and cocoa butter; Table 5) were analysed in order to know their exact nutritional values to allow the formulation of the experimental diets. All the raw ingredients were measured and mixed with warm tap water (28 °C) to achieve 70% moisture content in the diets. After the preparation, a sample of each experimental diet and GA (150 g) were chemically analysed (Table 6).

**Table 5:** Chemical composition (g/100 g as is) and gross energy of the semi-purified ingredients used for the 5 experimental diets.

Ingredients	Casein	Cocoa butter	Wheat starch
DM	92.05	99.81	91.13
CP	92.61	0.32	2.56
EE	0.30	99.62	0.03
Ash	4.05	0.05	0.63
aNDFom	0.00	0.00	0.00
NSC^1^	2.94	0.00	71.24
GE, MJ/kg	23.52	39.82	17.43

DM: dry matter, CP crude protein, EE ether extract, aNDFom amylase neutral detergent fiber organic matter, NSC non-structural carbohydrates, GE gross energy.

^a^Values are reported as mean of duplicate analyses.

^1^Calculated as 100−[(100−DM) + CP + EE + Ash + aNDFom].

**Table 6:** Ingredients (g/kg, as is), chemical composition (g/100 g on DM) and gross energy (MJ/kg, on DM) of the 5 experimental substrates and the Gainesville diet.

Items	L1	L1.5	L2.5	L3.5	L4.5	GA
Starch	833	827.5	822.5	817	812	-
Casein	160	160	160	160	160	-
Cocoa butter	7	12.5	17.5	23	28	-
Corn	-	-	-	-	-	200
Alpha-alpha	-	-	-	-	-	300
Wheat bran	-	-	-	-	-	500
Total	1000	1000	1000	1000	1000	1000
**Chemical composition**^**a**^						
DM	91.21	91.25	91.45	91.64	91.51	82.77
CP	17.36	17.39	17.51	17.31	17.47	15.28
EE	0.94	1.46	2.54	3.50	4.65	2.94
Ash	1.07	1.06	1.03	1.04	1.04	6.46
aNDFom	0.46	0.89	0.43	0.28	0.51	0.16
NSC^1^	80.17	79.20	78.49	77.87	76.33	75.16
GE	15.45	15.62	15.92	16.01	16.06	18.92

*Legend*: DM: dry matter, CP crude protein, EE ether extract, aNDFom amylase neutral detergent fiber organic matter, NSC non-structural carbohydrates, GE gross energy.

^a^Values are reported as mean of duplicate analyses.

^1^Calculated as 100 – (CP + EE + Ash + aNDFom).

### Growth trial

One-day-old larvae were provided by a European insect producer (Hermetia Baruth GmbH Baruth/Mark, Germany), and were reared for 5 days on GA in a climatic chamber under controlled environmental conditions (T°: 28 ± 0.5 °C; RH: 70 ± 5%; 0:24 L:D), using plastic boxes (19 cm × 13 cm × 6 cm) cover by a lid with nylon grid to allow air exchange.

To increase homogeneity in the sample, 6-days-old larvae were sieved with a mesh diameter of 0.8 mm, and only the larvae which passed through the sieve were used. To obtain 6 replicates for each diet, 20 groups of 5 larvae were weighed (Kern & Sohn GmbH; Balingen, Germany; d = 0.001) and then, if the standard deviation of the larvae weight was less than 0.02, they were pooled together (100 larvae/replicate).

Each replicate consisted of a plastic box (14 × 14 × 7 cm) filled with 180 g of substrate (1.8 g/larvae) and covered with a nylon grid to allow air exchange. Larvae were directly distributed on the substrate after the acclimation of the latter in the climatic chamber, in order to avoid a thermal shock for the formers. The plastic boxes were kept in the same climatic chamber where the 1-day-old larvae were reared.

### Larval stage

Each replica was observed daily to monitor the evolution of the substrate, and replicates were randomly redistributed to avoid bias from temperature being slightly higher at the top of the shelves in the climatic chamber. Every 4 days from the first day of the trial, 30 larvae were randomly taken from each replica by carefully avoiding the substrate removal. The BSFL were washed, gently dried with a paper tissue, and weighed. As the measure was not destructive, BSFL were put back in their box. This operation was repeated up until a replica reached 40% of the BSFL turning into prepupae, the moment in which the larval stage was considered ended in accordance to Tomberlin et al.^33^. To know exactly when a replica reached 40% of prepupae, all the replicates were daily checked after the first prepupa was observed. At the end of larval stage, the total number of larvae and prepupae was assessed for the survival calculation and the time interval from larva to prepupa was recorded (L-Pp, days). For the evaluation of the prepupae growth performance, 30 prepupae were randomly taken, washed, gently dried with paper tissue, weighed (Kern & Sohn GmbH; Balingen, Germany; d = 0.001), and put back in their box with the rest of the larvae to let them reach the pupal stage.

### Chemical analysis

The semi-purified ingredients, the diets and the larvae were stored at -20 °C. The larvae were ground (Retsch, GM 200) as frozen and freeze-dried. For the proximate composition analysis, the dry matter (DM; AOAC #934.01), the crude protein (CP; AOAC #984.13; conversion factor for ingredients and diets N × 6.25, for larvae N × 4.67^47^) and the ash (AOAC #942.05) were determined by the International AOAC^48^, the ether extract (EE; AOAC #2003.05) by the International AOAC^49^, and the amylase neutral detergent fiber and organic matter (aNDFom) by Mertens^50^. The non-structural carbohydrates (NSC) were calculated as difference with the other nutrients. The gross energy (GE) was analysed using an adiabatic calorimetric bomb (C7000; IKA, Staufen, Germany).

### Pupal and adult stages

To collect 23 pupae (characterized by their rigidity and immobility) per replicate for the determination of pupa and adult parameters, each replica was daily checked. When a pupa was found, it was cleaned without washing it, weighed, and then put in a transparent plastic “emergence box” (circular, 8 cm of diameter, 4 cm height; 1 pupa/box) with a perforated transparent plastic lid. The time interval from larva to pupa was also recorded (L–P, days).

Emergence box weight (EBW, g) was recorded before inserting the pupa. The emergence boxes were kept in the same climatic chamber used for rearing the larvae and were daily checked for the presence of flies. Once a fly emerged, the emergence box (with the puparium and the fly) was weighted (EBW_PF, g) to calculate the fly live weight (FLW, g) and the sex of the fly was assessed. The date of the emergence was recorded to evaluate the duration of the fly’s development (pupa-fly [P–F]). The box was put back in the climatic chamber until the death of the fly. Once the fly died, the emergence box was opened, the puparium (PW, g) and the dead fly (DFW, g) were weighed separately, and the sex of the fly checked a second time for accuracy. The DFW was used to calculate the weight reduction of the flies (WR, %). The fly death date was recorded in order to calculate the lifespan of the fly (FLS, days).

The formulas to calculate the parameters which were not directly measured are:

$$\mathrm{Fly\, Live\, Weight }\,\left(\mathrm{FLW},\mathrm{ g}\right)\, FLW= EBW\_PF-EBW-PW$$

$$\mathrm{Weight\, Reduction\, }(\mathrm{WR},\,\mathrm{ \%})\,\mathrm{ WR }=\mathrm{ FLW}\, -\left(\frac{DFW}{FLW} \times 100\right)$$

### Emergence rate and sex ratio

To assess the overall emergence rate (ER) and sex ratio (SR; female-to-male ratio), all the remaining larvae/prepupae of each replica were left untouched in the climatic chamber until fly emergence. Once all flies’ dead, the two parameters were calculated.

$$ER=\frac{n^\circ emerged\, flies\, \times100}{n^\circ pupae\, collected\, per\, treatment}$$

### Larvae descriptive nutritional composition

Simultaneously to the growth trial, a larger scale experiment was set up with the aim of collecting the larvae samples for the chemical composition. A total of 2 replicates per each dietary treatment were performed in a 23 × 30 × 9 cm box with a lid with perforated nylon to allow the air circulation. Each replica consisted of 1500 6-day-old larvae sieved with a 0.8 mm mesh and fed 2700 g of experimental diet (1.8 g/larva). The number of larvae was estimated by sampling (3 samples taken, with a coefficient of variation among the samples < 10%). The BSFL were left feeding on the experimental diets under controlled environmental conditions (T°: 28 ± 0.5 °C; RH: 70 ± 5%; 0:24 L:D). The boxes were daily observed and, when the first prepupa was detected, the larvae were collected, washed, gently dried with paper tissue, and inactivated at −80 °C. Larvae samples were processed and analysed using the method listed in the chemical analysis paragraph.

### Statistical analysis

Since the GA was considered only as environmental control, it was excluded from the statistical analysis (data reported in the Supplementary Information). Data were analysed using the IBM SPSS Statistics software (V20.0.0.). The statistical unit for the parameters recorded during the larval, prepupal, pupal and adult stages was the individual, with the exception of ER and SR, calculated as percentage or ratio of the total, in which it was the replicate. Shapiro–Wilk test was performed in order to evaluate the normality of the residuals, while the assumption of equal variances was assessed by Levene’s homogeneity of variance test. A generalized linear mixed model (GLMM) with a gamma probability distribution (nonlinear link function [log]) was fitted for the larvae weight and adult parameters, with two fixed factors being considered (diet/time for the larvae and diet/sex for the flies, plus their interaction). If the interaction was not significant, a likelihood-ratio was carried out and, when necessary, a model simplification by removing it was applied. In order to account the repeated measurements during the time, replicates were included in the model as a random effect. The interactions between the levels of the fixed factors were evaluated by means of pairwise contrasts.

Since in prepupal and pupal stages the replicate was considered as random effect, an analogous GLMM was applied. The ER and SR were, instead, analysed by one-way ANOVA test (post-hoc test: Tukey). The results were expressed as mean and pooled standard error of the mean (SEM). The level of significance considered was < 0.05.

## Supplementary Information

Supplementary Information.

## Data Availability

The datasets generated during and/or analysed during the current study are available from the corresponding author on reasonable request.
