# Supplementary material for: Determination of lipid requirements in black soldier fly through semi-purified diets
Source: Sci Rep. 2022 Jun 28;12:10922. doi: 10.1038/s41598-022-14290-y (PMC9239991; doi:10.1038/s41598-022-14290-y)
Supplement: Supplementary file 1 — Supplementary Information. [file 41598_2022_14290_MOESM1_ESM.pdf]

Determination of lipid requirements in black soldier fly through semi-purified diets

S. Bellezza Oddon[a], I. Biasato[a]*, A. Resconi[a], L. Gasco[a]
[a]Department of Agricultural, Forest and Food Sciences, University of Turin, Italy
* Corresponding author: ilaria.biasato@unito.it
E-mail addresses: sara.bellezzaoddon@unito.it; laura.gasco@unito.it; andrea.resconi@unito.it; ilaria.biasato@unito.it

Supplementary Information

Gainesville diet

Data regarding the larva, prepupa, pupa and adult parameters recorded on the GA are shown in Table 1, while in Table 2 is illustrated the larvae chemical composition.

Table 1. Effect of the environmental control on BSF life history traits. L-Pp larva-prepupa, *L-P* larva-pupa, *WR* weight reduction, *FLW* fly live weight, *PW* puparium weight, *P-F* pupa-fly, *FLS* fly lifespan, *ER* emergence rate, *SR* sex ratio.

**Larvae (day old)**	**Weight (g)**
6	0.088
10	0.099
14	0.156
18	0.183
Survival rate (%)	96
**Prepupae**	
Weight (g)	0.165
L-Pp duration (day)	8.0
**Pupae**	
Weight (g)	0.149
L-P duration (day)	13.62
**Adult**	
WR (%)	35.68
FLW (g)	0.097
PW (g)	0.188
P-F duration (day)	7.35
FLS (day)	7.22

ER (%)	97.66
SR	0.92

Table 2. Chemical composition (on DM) of the larvae reared on GA. DM: dry matter, CP crude protein, EE ether extract, GE gross energy.
[a]Values are reported as mean of duplicate analyses

**Items**	**Larvae (% on DM)**
DM	22.83
CP	54.26
EE	13.58
Ash	10.88
GE	22.25
